# Optimizing Compassion Training in Medical Trainees Using an Adjunct mHealth App: A Preliminary Single-Arm Feasibility and Acceptability Study

**DOI:** 10.2196/60670

**Published:** 2024-11-26

**Authors:** Jennalee S Wooldridge, Emily C Soriano, Gage Chu, Anaheed Shirazi, Desiree Shapiro, Marta Patterson, Hyun-Chung Kim, Matthew S Herbert

**Affiliations:** 1 Mental Health Service VA San Diego Healthcare System San Diego, CA United States; 2 Department of Psychiatry University of California San Diego San Diego, CA United States; 3 Center of Excellence for Stress and Mental Health (CESAMH) San Diego, CA United States; 4 Scripps Whittier Diabetes Institute San Diego, CA United States; 5 University of California San Diego Center for Mindfulness San Diego, CA United States

**Keywords:** mobile phone, compassion, empathy, mHealth, mobile health, medical student, medical resident, mHealth app, app, medical trainee, training, feasibility, acceptability, pilot, mindfulness, self-compassion, smartphone app, compassion, applicability

## Abstract

**Background:**

While structured compassion training programs have shown promise for increasing compassion among medical trainees, a major challenge is applying the concepts and practices taught during the program into the complex, dynamic, time-pressured, and often hectic hospital workplace.

**Objective:**

The purpose of this pilot study was to examine the feasibility, acceptability, and preliminary effects of Compassion Coach, a mobile health (mHealth) smartphone app designed to bolster a 6-week mindfulness and self-compassion training program for medical trainees.

**Methods:**

In Compassion Coach, notifications to remind, encourage, and measure the perceived impact of informal mindfulness and compassion practices taught during the program were delivered at 7 AM, 12 PM, and 7 PM, respectively, 3 times per week over the course of the training program. The app also contained a library of guided audio formal mindfulness and compassion practices to allow quick and easy access. In this pilot study, we collected data from 29 medical students and residents who downloaded Compassion Coach and completed surveys assessing perceived effectiveness and acceptability. Engagement with the Compassion Coach app was passively tracked through notification response rate and library resource access over time.

**Results:**

The average response rate to notifications was 58% (SD 29%; range 12%-98%), with a significant decline over time (*P*=.009; odds ratio 0.98, 95% CI 0.96-0.99). Across all participants and occasions, the majority agreed the informal practices prompted by Compassion Coach helped them feel grounded and centered (110/150, 73%), improved compassion (29/41, 71%), reduced burnout (106/191, 56%), and improved their mood (133/191, 70%). In total, 16 (55%) of the 29 participants accessed guided audio recordings on average 3 (SD 3.4) times throughout the program. At the posttreatment time point, most participants (13/18, 72%) indicated that Compassion Coach helped them engage in compassion practices in daily life, and half (9/18, 50%) indicated that Compassion Coach helped improve interactions with patients.

**Conclusions:**

Overall, preliminary results of Compassion Coach are encouraging and suggest the integration of a smartphone app with an ongoing mindfulness and self-compassion training program may bolster the effects of the program on medical trainees. However, there was variability in engagement with Compassion Coach and perceived helpfulness. Additional research is indicated to optimize this novel mHealth approach and conduct a study powered to formally evaluate effects.

## Introduction

Compassion and self-compassion in health care have the potential to directly benefit patients (eg, greater patient satisfaction and engagement with treatment) and medical professionals (eg, greater well-being and reduced burnout) [1,2]. Yet, some reports suggest that levels of compassion may be suboptimal in medical professionals [3,4], exacerbated by the challenges of the COVID-19 pandemic [5]. Thus, there is a need to bolster and maintain compassion among medical professionals, particularly medical trainees in the early stages of their careers.

In response to this need, compassion training programs for medical professionals and trainees have been developed [6-9]. These programs often include formal and informal practices assigned as homework to optimize impact. Formal practices refer to meditation practices (eg, mindfulness meditation and contemplative compassion meditation) [10]. Informal practices refer to more brief exercises focused on increasing mindfulness and compassion in daily life (eg, mindful eating and internal acts of compassion) [11]. Although engagement in formal practice is associated with beneficial outcomes [12-14], it may be particularly challenging for medical trainees given the time demands of formal practice [15,16] and ultimately less important than the ability to apply informal compassion practices to the complex, dynamic, time-pressured, and often hectic clinical workplace. However, engagement in informal practices by medical professionals or trainees has not been well studied.

One potential strategy to improve engagement in formal and informal practices in medical trainees is by using smartphone apps to augment traditional classroom-based compassion training. This approach capitalizes on the availability of smartphones to reinforce material from compassion programs with minimal impact on their schedules. This study aims to examine the feasibility, acceptability, and preliminary effects of Compassion Coach, a mobile health (mHealth) smartphone app designed to encourage and measure formal and informal compassion practices associated with a 6-week mindfulness and self-compassion training program for medical trainees.

## Methods

### Participants

Participants (N=29) were 15 medical students and 14 medical residents, primarily between the ages of 30 and 35 years (17/29, 59%) within the University of California San Diego (UCSD) and Charles Drew University (CDU) medical schools. We targeted a sample size of 25-30 participants to meet practical and budgetary constraints while still being able to evaluate feasibility in preparation for a larger trial [17]. Recruitment occurred in October of 2022. Data was collected from November 2022 to March 2023. Over half identified as female (n=17, 59%) and 1 (3%) participant identified as nonbinary. Most participants identified as Asian or Asian American (n=11, 38%) or White (n=10, 35%). Of the remaining participants, 10% (n=3) identified as another race, 7% (n=2) identified as Black or African American, 7% (n=2) preferred not to say, and 3% (n=1) identified as American Indian or Alaska native. Across all races, 24% (n=7) identified as Hispanic or Latinx.

### Ethical Considerations

The study was approved by the UCSD institutional review board, and informed consent was obtained at enrollment. Adults were eligible to participate in the study if they were UCSD or CDU medical students or residents and owned a smartphone. Participants were informed that their responses were confidential. All participant data were deidentified before analysis. Participants were compensated up to US $80 for their time and effort in the study. Analyses conducted are covered by our institutional review board and consent form.

### Procedure

All participants were enrolled in a 6-week Introduction to Mindfulness and Self-Compassion training program offered by the UCSD Center for Mindfulness. The program was modified by program facilitators and research personnel to focus on relevant aspects of medical training and provision (eg, grounding techniques before medical charting and performing a brief mindfulness exercise before patient care). At the end of each session, participants were encouraged to engage in the Compassion Coach app’s formal and informal practices as homework. Formal compassion practices included a variety of guided audio contemplative meditations intended to help settle the mind, offer loving kindness to the self and others, and cultivate a greater understanding of suffering. Informal practices consisted of grounding exercises, intention setting, mindful savoring, wishing well to others, intentional acts of kindness, and reframing self-criticism with self-compassion (refer to Multimedia Appendix 1 for descriptions of informal practices).

### Compassion Coach App Content

**Figure 1:**
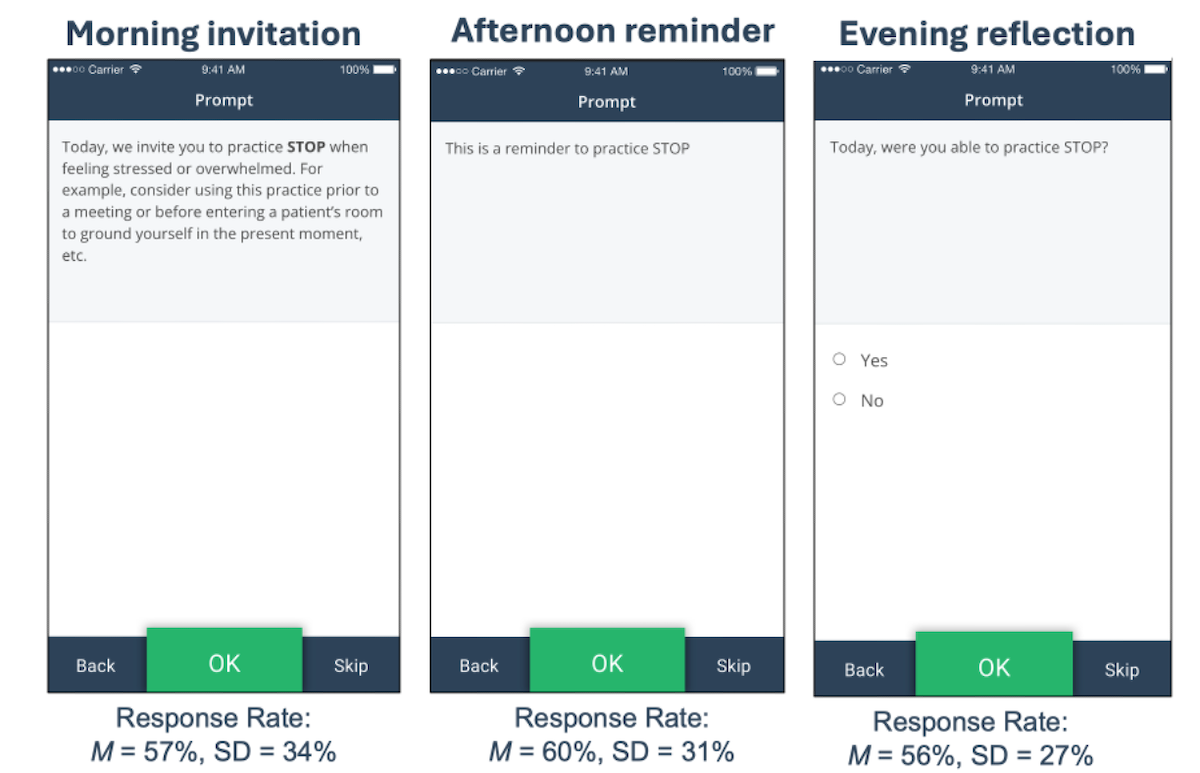
Screenshots showing examples of app prompts. M: mean.

Compassion Coach consisted of a “Morning invitation” to encourage engagement in informal practice introduced at the previous program session, an “Afternoon reminder” to remind the participant to engage in the practice, and an “Evening reflection” to assess practice engagement and the perceived impact of participation on mindfulness, compassion, burnout, and mood (refer Figure 1 for examples). If participants indicated they were not able to complete the practice for that day, they were prompted for an explanation. These 3 prompts were delivered at 7 AM, 12 PM, and 7 PM, respectively, 3 times per week during the mindfulness and self-compassion training program.

The app also contained a library of guided audio formal compassion practices to allow quick and easy access [18]. Finally, a brief posttraining survey was administered after the completion of the program and use of Compassion Coach. Regarding compensation, participants were paid US $20 for initially downloading the app, US $1 for each prompt that was opened, and an additional US $6 for completing the posttraining survey for up to US $80 total compensation.

### Measures

Feasibility was passively tracked by the software app and assessed through (1) the rate of response to Compassion Coach prompts and (2) the frequency of accessing the library of guided compassion practices. Response to prompts was defined as opening the morning, afternoon, or evening prompt notification.

Acceptability was assessed with three questions in the posttraining survey: (1) “Compassion Coach helped me engage in compassion practices in my daily life,” (2) “Overall, how satisfied were you with Compassion Coach?” and (3) “Compassion Coach helped improve my interactions with patients.” Response options ranged from 0 (strongly disagree) to 10 (strongly agree). To enhance interpretability, responses 0-4 were considered “disagree,” a response of 5 was considered “neutral,” and responses 6-10 were considered “agree.”

The preliminary effectiveness of Compassion Coach was assessed in the app’s evening reflection questions. Evening reflection questions included “Today, were you able to practice [WEEKLY PRACTICE]” with a yes or no response option; if yes, “This practice [helped me feel grounded and centered or improved my sense of self-compassion or improved my mood or reduced my sense of burnout]” (0 [Strongly disagree] to 10 [Strongly agree] slider); and if no, “What got in the way?” (not enough time or no opportunity or did not feel like it or forgot or other [TEXT]). Responses 0-4 were categorized as “disagree,” a response of 5 was categorized as “neutral,” and responses 6-10 were categorized as “agree.”

### Data Analysis

Data analysis was performed in SPSS (IBM Corp; version 29) and Mplus (Muthén & Muthén, version 8.7). Mplus handles missing data using full information maximum likelihood. At the person level, mean response rates to Compassion Coach prompts were computed separately for morning invitations, afternoon reminders, evening reflections, and all prompts combined. Person-level averages were also computed for responses to the 4 self-report items included in the evening reflections. Descriptive statistics (mean, SD, and frequencies) were computed for all of these person-level means, sociodemographic variables, and posttreatment survey responses. The intraclass correlations were computed for the 4 evening reflection items to estimate the relative proportion of between-person variability to total (between- and within-person) variability. Using 2-level multilevel modeling (prompts nested within participants), we examined whether response rates significantly changed over time, which may mark assessment-related reactivity or fatigue. Specifically, time (represented in days) was entered as a predictor of prompt response (yes or no) in a logistic regression model. Person-level descriptives and frequencies were estimated to determine how many participants engaged in formal practices, the average number of times participants accessed formal practices, and the breakdown by formal practice type. In the *Results* section, between-person statistics are denoted by a subscript (_BP_), whereas the within-person statistics are denoted by a subscript (_WP_).

## Results

The average response rate across all Compassion Coach prompts was 58% (31/54 prompts; SD 29%, rounded to integers), with little variability in response rate based on prompt type: morning invitation 57% (10/18; SD 34%, rounded to integers), afternoon reminder 61% (11/18; SD 31%, rounded to integers), and evening reflection 56% (10/18; SD 27%, rounded to integers). On average, each participant completed 6 to 8 entries for the evening reflections concerning mindfulness, burnout, and mood, with an average of 3 entries for the evening reflection item regarding self-compassion. In the 2-level multilevel model examining linear change in response rate over time, results indicated that time (days) significantly predicted overall prompt response rate over time, (*P*=.009; odds ratio 0.98, 95% CI 0.96-0.99), indicating that as time passed, the odds of responding decreased.

On the original 0 (strongly disagree) to 10 (strongly agree) scale, participants generally were neutral or agreed that practices reduced their sense of burnout (mean 5.2, SD_BP_ 1.4, SD_WP_ 1.2), improved their mood (mean 5.9, SD_BP_ 1.2, SD_WP_ 1.2), helped them feel grounded and centered (mean 6.5, SD_BP_ 0.94, SD_BP_ 1.4), and improved their sense of compassion (mean 6.4, SD_BP_ 1.6, SD _WP_ 1.7). When categorized as “agree,” “neutral,” or “disagree” and combining all total entries, the breakdown for each item was as follows: (1) burnout: 56% (106/191) “agreed,” 22% (41/191) “neutral,” and 23% (44/191) “disagreed”; (2) mood: 70% (133/191) “agreed,” 18% (34/191) “neutral,” and 13% (24/191) “disagreed”; (3) grounded and centered: 73% (110/150) “agreed,” 17% (25/150) “neutral,” and 10% (15/150) “disagreed”; and (4) compassion: 71% (29/41) “agreed,” 12% (5/41) “neutral,” and 17% (7/41) “disagreed.”

Across participants and time points, the average response to the evening reflection item, “today, were you able to practice...” was a mean of 0.55 (SD 0.33), indicating that it was endorsed more than half the time. The intraclass correlation for this item was .20, indicating that on average, the responses to this item varied more within-person over time (80% of total variability) than between-person (20% of total variability). Also, across participants and time points, the endorsed reasons for not practicing exercises were as follows (from most to least frequent): 52% (58/112) “forgot,” 27% (30/112) “did not have the opportunity,” 26% (29/112) “did not have time,” 11% (12/112) “other,” and 9% (10/112) “did not feel like it.”

The formal practice data revealed that 16 (55%) of the 29 participants accessed guided audio recordings, on average 3 times (SD 3.4) throughout the program. The most commonly accessed practice types were self-compassion (14/46, 30%), soften-soothe-allow (9/46, 20%), body scan (8/46, 17%), compassion with equanimity (7/46, 15%), awareness of breath (6/46, 13%), affectionate breathing (1/46, 2%), and open awareness (1/46, 2%).

Among those who completed the posttreatment survey (n=18), 72% (n=13) “agreed” that Compassion Coach helped engage in compassion in daily life, 50% (n=9) were “satisfied” with Compassion Coach, and 50% (n=9) agreed that Compassion Coach helped improve interactions with patients. Across the 3 items, 4-5 (22%-25%) participants had negative feedback about Compassion Coach by reporting they “disagreed” and were “dissatisfied.”

## Discussion

### Principal Findings

This study examined the feasibility, acceptability, and preliminary effects of Compassion Coach, an mHealth app developed as an adjunctive to a 6-week mindfulness and self-compassion training program for medical trainees. Overall, results suggest Compassion Coach was feasible and acceptable among medical trainees (students and residents). However, there was substantial variability in both engagement and perceived impact of Compassion Coach. This suggests different medical trainees may benefit from different strategies for increasing compassion. Future work should focus on tailoring Compassion Coach according to participant characteristics and preferences.

Response rates were acceptable but lower than average for intensive longitudinal studies [19]. Engagement with Compassion Coach declined over the 6-week intervention, suggesting that additional modifications are needed to improve engagement, particularly during the latter half of the program. Just over half the time, participants endorsed participating in informal practices prompted by Compassion Coach. Forgetting, followed by not having the opportunity, were the most common reasons for being unable to practice. Individually tailored time windows for optimal delivery of prompts and reminders may improve engagement rates.

Participants were encouraged to engage in formal practices using the Compassion Coach app. However, only 55% (16/29) of participants accessed guided-audio formal practices over the 6-week program. We did not collect feedback on barriers to engagement in formal practices, although it is possible that participants struggled to find the time to dedicate to formal practice, which has been found in previous studies examining mindfulness programs in medical trainees [15,16]. Although we were primarily interested in informal practices that can be used in proximity to patient interactions, adherence to formal compassion practice is associated with several positive outcomes [12-14,20]. Future research is encouraged to identify strategies to promote both formal and informal compassion practice in medical professionals.

Most participants agreed Compassion Coach helped improve feeling grounded and centered, compassion, burnout, and mood. These initial results are promising for Compassion Coach’s benefits. Notably, participants were least likely to agree Compassion Coach helped improve burnout, a key issue in medical trainees [21]. More targeted practices to address burnout may improve the benefit of the app. However, burnout may also be driven by larger systematic factors such as work demands and poor work environment [22], suggesting the need for organizational interventions in addition to medical trainee intervention.

### Conclusion

There were important limitations to this study. Biases associated with self-reporting and retrospection may be present. In addition, participants who did not complete the posttreatment survey may differ from those who completed it in terms of their perceived benefit. Because of the pilot design, this study had a small sample size. Future larger studies would enable the comparison of different levels of training, specialties, and sociodemographic characteristics. In addition, future qualitative research may help with gaining insight into factors motivating engagement with the app. This study also has notable strengths, including the examination of a tailored adjunct mHealth app with mostly positive impacts on medical trainees. Feasibility and acceptability were largely supported, although additional refinement of Compassion Coach may be indicated to more thoroughly tailor to individual characteristics and preferences.

## Appendix

Multimedia Appendix 1 Informal mindfulness and compassion practices.

## Abbreviations

mHealth: mobile health
UCSD: University of California San Diego

## Data Availability

The datasets generated during and/or analyzed during this study are available from the corresponding author on reasonable request.
