# Supplementary material for: Optimizing Compassion Training in Medical Trainees Using an Adjunct mHealth App: A Preliminary Single-Arm Feasibility and Acceptability Study
Source: JMIR Form Res. 2024 Nov 26;8:e60670. doi: 10.2196/60670 (PMC11632279; doi:10.2196/60670)
Supplement: Multimedia Appendix 1 [file formative_v8i1e60670_app1.docx]

Informal Mindfulness and Compassion Practices

**Week 1: Soles of the feet**

Sensing the sensations at the soles of the feet as a way to anchor one's attention to the present moment. It can be easily incorporated throughout the day, during rounds or intake. Soles of the feet practice is an accessible practice to bring your attention back to the present moment.

**Week 2: 3-minute breathing space**

A brief, 3-minute meditation where you start by recognizing current thoughts, feelings, and body sensations as they are. With this awareness, shifting the attention to the sensations of the breath and then to the physical sensations in the body as a whole. This allows the mind to step out of automatic, habitual patterns of the mind and create space around one's present moment experience.

**Week 3: STOP practice**

STOP is an acronym.

S: Stop what you are doing

T: Take a couple of deep breaths

O: Observe what is happening in the mind and body

P: Proceed with wise efforts.

This practice can remind us how to respond to difficult, stressful situations more mindfully.

**Week 4: Self-compassion break**

This exercise incorporates the three components of self-compassion. Practice by recalling a mildly stressful situation to mind, and as you feel the sensations of stress in the mind and body, we practice being aware of the suffering(mindfulness), acknowledge suffering a part of life (common humanity), and finally offer yourself kindness phrases and soothing/supportive touch to cultivate self-compassion (kindness).

**Week 5: Labeling emotions**

Label emotions when you feel stressed – “Anxiety is arising”, “Frustration is here”, “Sadness is present”. Allow it to be there with perspective. Recognize it doesn't define you or the moment, it is just here. Difficult emotions are part of being human. Offer kindness to yourself because it's difficult. Labeling pleasant emotions is also good practice to know what you're feeling moment by moment.

**Week 6: Compassion with equanimity**

 Equanimity is a maintained state of balance in the presence of pleasant and unpleasant emotions. This exercise combines a breathing exercise ("Breathing compassion in for me and out for you") with equanimity phrases, and soothing/supportive touch. It cultivates compassion for the practitioner while maintaining a kind and caring connection with the patient.

Content of Morning Invitations, Afternoon Reminders, and Evening Reflections

| **Week 1 – Mon, Wed, and Fri** | |
| --- | --- |
| **Topic** | Week 1: Soles of the Feet |
| **Morning Invitation** | “Soles of the Feet” |
| Prompt sent at 7:00AM | “Today, we invite you to practice **Soles of the Feet**. Try using this practice prior to logging into Epic, transitioning between patients’ charts, prior to starting a meeting to ground yourself in the present moment, and/or before entering a patient’s room for interviews.” |
| **Afternoon Reminder** | “Week 1 Afternoon Reminder” |
| Prompt sent at 12:00PM | “This is a reminder to practice Soles of the Feet” |
| **Evening Reflection** | “Week 1 PM Reflection” |
| Prompt sent at 7:00PM | “Today, were you able to practice Soles of the Feet?” (Yes/No)  If yes 🡪 “This practice helped me feel grounded and centered?”  [0 (Strongly disagree) -10 (Strongly agree) slider]  If yes 🡪 “This practice improved my mood.”  [0 (Strongly disagree) -10 (Strongly agree) slider]  If yes 🡪 “This practice reduced my sense of burnout.”  [0 (Strongly disagree) -10 (Strongly agree) slider]  If no 🡪 “What got in the way?” not enough time/no opportunity/did not feel like it/forgot/other (TEXT) |
| **Week 2 – Mon, Wed, and Fri** | |
| **Topic** | Week 2: 3-minute Breathing Space |
| **Morning Invitation** | 3-minute Breathing Space |
| Prompt sent at 7:00AM | “Today, we invite you to practice **3-minute Breathing Space**. Try using this practice when transitioning from patient interviews to note documentation and/or after interviewing patients in the morning and before morning rounds.” |
| **Afternoon Reminder** | “Week 2 Afternoon Reminder” |
| Prompt sent at 12:00PM | “This is a reminder to practice 3-minute Breathing Space” |
| **Evening Reflection** | “Week 2 PM Reflection” |
| Prompt sent at 7:00PM | “Today, were you able to practice 3-minute Breathing Space?” (Yes/No)  If yes 🡪 ”This practice helped me feel grounded and centered?”  [0 (Strongly disagree) -10 (Strongly agree) slider]  If yes 🡪 “This practice improved my mood.”  [0 (Strongly disagree) -10 (Strongly agree) slider]  If yes 🡪 “This practice reduced my sense of burnout.”  [0 (Strongly disagree) -10 (Strongly agree) slider]  If no 🡪 “What got in the way?” not enough time/no opportunity/did not feel like it/forgot/other (TEXT) |
| **Week 3 – Mon, Wed, and Fri** | |
| **Topic** | Week 3: STOP Practice |
| **Morning Invitation** | STOP Practice |
| Prompt sent at 7:00AM | “Today, we invite you to practice **STOP**. Try using this practice prior to logging into Epic, transitioning between patients’ charts, prior to starting a meeting to ground yourself in the present moment, and/or before entering a patient’s room for interviews.” |
| **Afternoon Reminder** | “Week 3 Afternoon Reminder” |
| Prompt sent at 12:00PM | “This is a reminder to practice STOP” |
| **Evening Reflection** | “Week 3 PM Reflection” |
| Prompt sent at 7:00PM | “Today, were you able to practice STOP?” (Yes/No)  If yes 🡪 ”This practice helped me feel grounded and centered?”  [0 (Strongly disagree) -10 (Strongly agree) slider]  If yes 🡪 “This practice improved my mood.”  [0 (Strongly disagree) -10 (Strongly agree) slider]  If yes 🡪 “This practice reduced my sense of burnout.”  [0 (Strongly disagree) -10 (Strongly agree) slider]  If no 🡪 “What got in the way?” not enough time/no opportunity/did not feel like it/forgot/other (TEXT) |
| **Week 4 – Mon, Wed, and Fri** | |
| **Topic** | Week 4: Self-compassion Break |
| **Morning Invitation** | Self-compassion Break |
| Prompt sent at 7:00AM | “Today, we invite you to practice the **Self-compassion Break**. After morning interviews and rounds, try to identify thoughts that follow these patterns: “I should have said..” or “I should not have said…” and practice Self-compassion Break during lunch or at end of your workday.” |
| **Afternoon Reminder** | “Week 4 Afternoon Reminder” |
| Prompt sent at 12:00PM | “This is a reminder to practice Self-compassion Break” |
| **Evening Reflection** | “Week 4 PM Reflection” |
| Prompt sent at 7:00PM | “Today, were you able to practice Self-compassion Break?” (Yes/No)  If yes 🡪 ”This practice improved my sense of self-compassion”  [0 (Strongly disagree) -10 (Strongly agree) slider]  If yes 🡪 “This practice improved my mood.”  [0 (Strongly disagree) -10 (Strongly agree) slider]  If yes 🡪 “This practice reduced my sense of burnout.”  [0 (Strongly disagree) -10 (Strongly agree) slider]  If no 🡪 “What got in the way?” not enough time/no opportunity/did not feel like it/forgot/other (TEXT) |
| **Week 5 – Mon, Wed, and Fri** | |
| **Topic** | Week 5: Labeling Emotions |
| **Morning Invitation** | Labeling Emotions |
| Prompt sent at 7:00AM | “Today, we invite you to practice the **Labeling Emotions**. Practice the labeling emotions technique prior to a psychiatric hearing, writing the treatment plan section of your note, after morning rounds and having received feedback and/or after you respond to code greens or psychiatric emergencies.” |
| **Afternoon Reminder** | “Week 5 Afternoon Reminder” |
| Prompt sent at 12:00PM | “This is a reminder to practice Labeling Emotions” |
| **Evening Reflection** | “Week 5 PM Reflection” |
| Prompt sent at 7:00PM | “Today, were you able to practice Labeling Emotions?” (Yes/No)  If yes 🡪 “This practice improved my sense of self-compassion”  [0 (Strongly disagree) -10 (Strongly agree) slider]  If yes 🡪 “This practice improved my mood.”  [0 (Strongly disagree) -10 (Strongly agree) slider]  If yes 🡪 “This practice reduced my sense of burnout.”  [0 (Strongly disagree) -10 (Strongly agree) slider]  If no 🡪 “What got in the way?” not enough time/no opportunity/did not feel like it/forgot/other (TEXT) |
| **Week 6 – Mon, Wed, and Fri** | |
| **Topic** | Week 6: Compassion Equanimity |
| **Morning Invitation** | Compassion Equanimity |
| Prompt sent at 7:00AM | “Today, we invite you to practice the **Compassion Equanimity**. Practice compassion equanimity either prior or after interviewing patients in the morning, after placing a patient on an involuntary hold, and/or after ordering involuntary administration of medications.”  Practice compassion equanimity when discharging a patient against medical advice.” |
| **Afternoon Reminder** | “Week 6 Afternoon Reminder” |
| Prompt sent at 12:00PM | “This is a reminder to practice Compassion Equanimity” |
| **Evening Reflection** | “Week 6 PM Reflection” |
| Prompt sent at 7:00PM | “Today, were you able to practice Compassion Equanimity?” (Yes/No)  If yes 🡪 “This practice improved my sense of self-compassion”  [0 (Strongly disagree) -10 (Strongly agree) slider]  If yes 🡪 “This practice improved my mood.”  [0 (Strongly disagree) -10 (Strongly agree) slider]  If yes 🡪 “This practice reduced my sense of burnout.”  [0 (Strongly disagree) -10 (Strongly agree) slider]  If no 🡪 “What got in the way?” not enough time/no opportunity/did not feel like it/forgot/other (TEXT) |
